# [(1,2,5,6-η)-Cyclo­octa-1,5-diene](1-ethyl-4-iso­butyl-1,2,4-triazol-5-yl­idene)(tri­phenyl­phosphane)iridium(I) tetra­fluorido­borate di­chloro­methane hemisolvate

**DOI:** 10.1107/S2414314624009416

**Published:** 2024-09-30

**Authors:** Timothy G. Lerch, Michael Gau, Daniel R. Albert, Edward Rajaseelan

**Affiliations:** a Department of Chemistry Millersville University,Millersville PA 17551 USA; b Department of Chemistry, University of Pennsylvania, Philadelphia, PA 19104, USA; Sunway University, Malaysia

**Keywords:** iridium, *N*-heterocyclic carbene, non-merohedral twin, crystal structure

## Abstract

A new triazole-based *N*-heterocyclic carbene Ir^I^ cationic complex with a tetra­fluorido­borate counter-anion and hemi-solvating di­chloro­methane, [Ir(C_8_H_12_)(C_8_H_15_N_3_)(C_18_H_15_P)]BF_4_·0.5CH_2_Cl_2_, has been synthesized and structurally characterized. There are two independent ion pairs in the asymmetric unit and one di­chloro­methane solvent mol­ecule per two ion pairs. The cationic complex exhibits a distorted square-planar conformation around the Ir^I^ atom, formed by a bidentate cyclo­octa-1,5,diene (COD) ligand, a tri­phenyl­phosphane ligand, and an *N*-heterocyclic carbene (NHC). There are several close non-standard H⋯F hydrogen-bonding inter­actions that orient the tetra­fluorido­borate anions with respect to the Ir^I^ complex mol­ecules. The complex shows promising catalytic activity in transfer hydrogenation reactions. The structure was refined as a non-merohedral twin, and one of the COD mol­ecules is statistically disordered.

## Abstract 2

A new triazole-based *N*-heterocyclic carbene cationic iridium(I) complex with a tetra­fluorido­borate counter-anion and hemi-solvating di­chloro­methane, [Ir(C_8_H_12_)(C_18_H_15_P)(C_8_H_15_N_3_)][BF_4_]·0.5CH_2_Cl_2_, has been structurally characterized.

## Graphical abstract

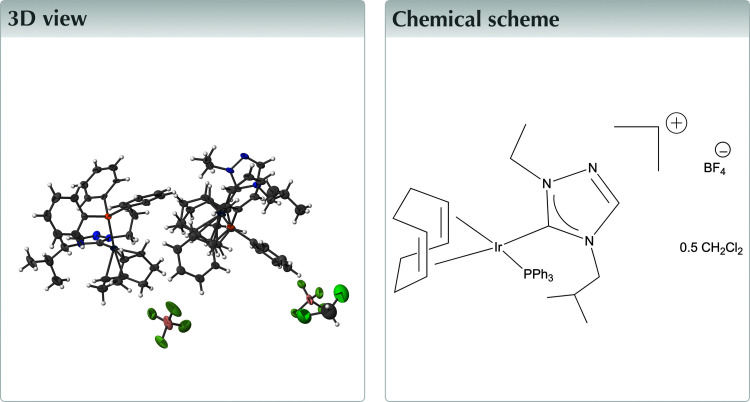

## Structure description

**Figure 1:**
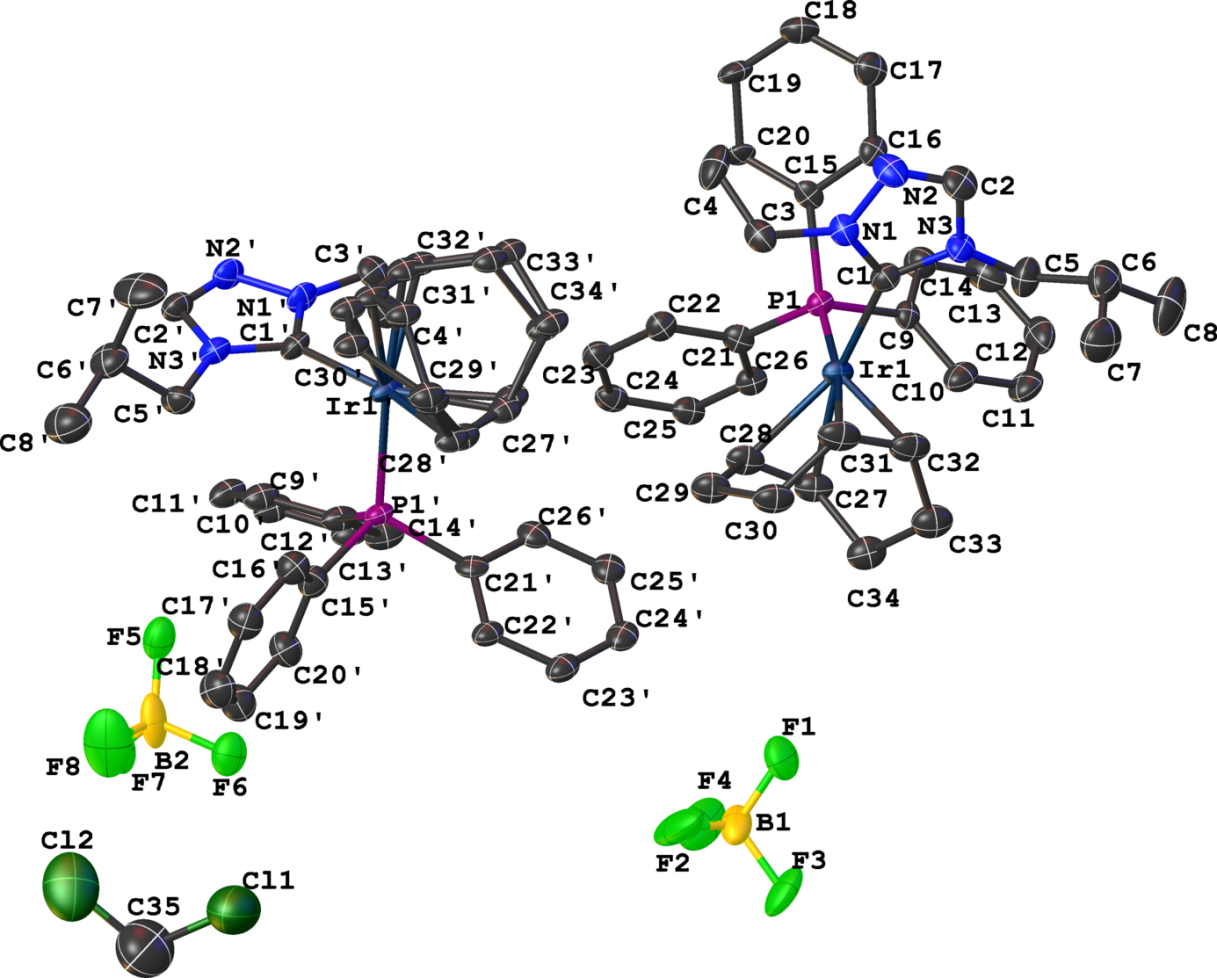
Mol­ecular entities of the title compound (**3**) showing the atom-labeling scheme and with displacement ellipsoids drawn at the 50% probability level.

*N*-heterocyclic carbenes (NHCs) have been considered alternatives to phosphanes as spectator ligands in homogeneous catalysis and can be tuned sterically and electronically by having different substituent groups on the nitro­gen atoms (Cazin, 2013 ▸; Diez-González *et al.*, 2009 ▸; Rovis & Nolan, 2013 ▸; Ruff *et al.*, 2016 ▸; Zuo *et al.*, 2014 ▸; Diez-González & Nolan, 2007 ▸; Gusev, 2009 ▸). Their catalytic activity in the transfer hydrogenation of ketones and imines has also been reported (Albrecht *et al.*, 2002 ▸; Gnanamgari *et al.*, 2007 ▸). Many imidazole- and triazole-based NHC rhodium and iridium complexes have been synthesized and structurally characterized (Herrmann *et al.*, 2006 ▸; Wang & Lin 1998 ▸; Chianese *et al.*, 2004 ▸). We continue to synthesize new imidazole- and triazole-based NHC complexes of rhodium and iridium, to study the effect of different substituents on the NHCs and the other ligands coordinated to the metal in transfer hydrogenation reactions (Nichol *et al.*, 2009 ▸, 2010 ▸, 2011 ▸, 2012 ▸; Idrees *et al.*, 2017*a* ▸,*b* ▸; Rood *et al.*, 2021 ▸; Rushlow *et al.*, 2021 ▸; Newman *et al.*, 2021 ▸; Castaldi *et al.*, 2021 ▸; Maynard *et al.*, 2023 ▸; Lerch *et al.*, 2024*a* ▸,*b* ▸,*c* ▸,*d* ▸). The asymmetric unit of the title complex, [Ir(C_8_H_12_)(C_18_H_15_P)(C_8_H_15_N_3_)][BF_4_] (**3**), comprises two independent Ir^I^ complex cations (*A* containing Ir1 and *B* containing Ir1′) and two independent tetra­fluorido­borate counter-anions, Fig. 1 ▸. One di­chloro­methane solvent mol­ecule per two ion pairs was also found in the crystal, Fig. 1 ▸.

**Figure 2:**
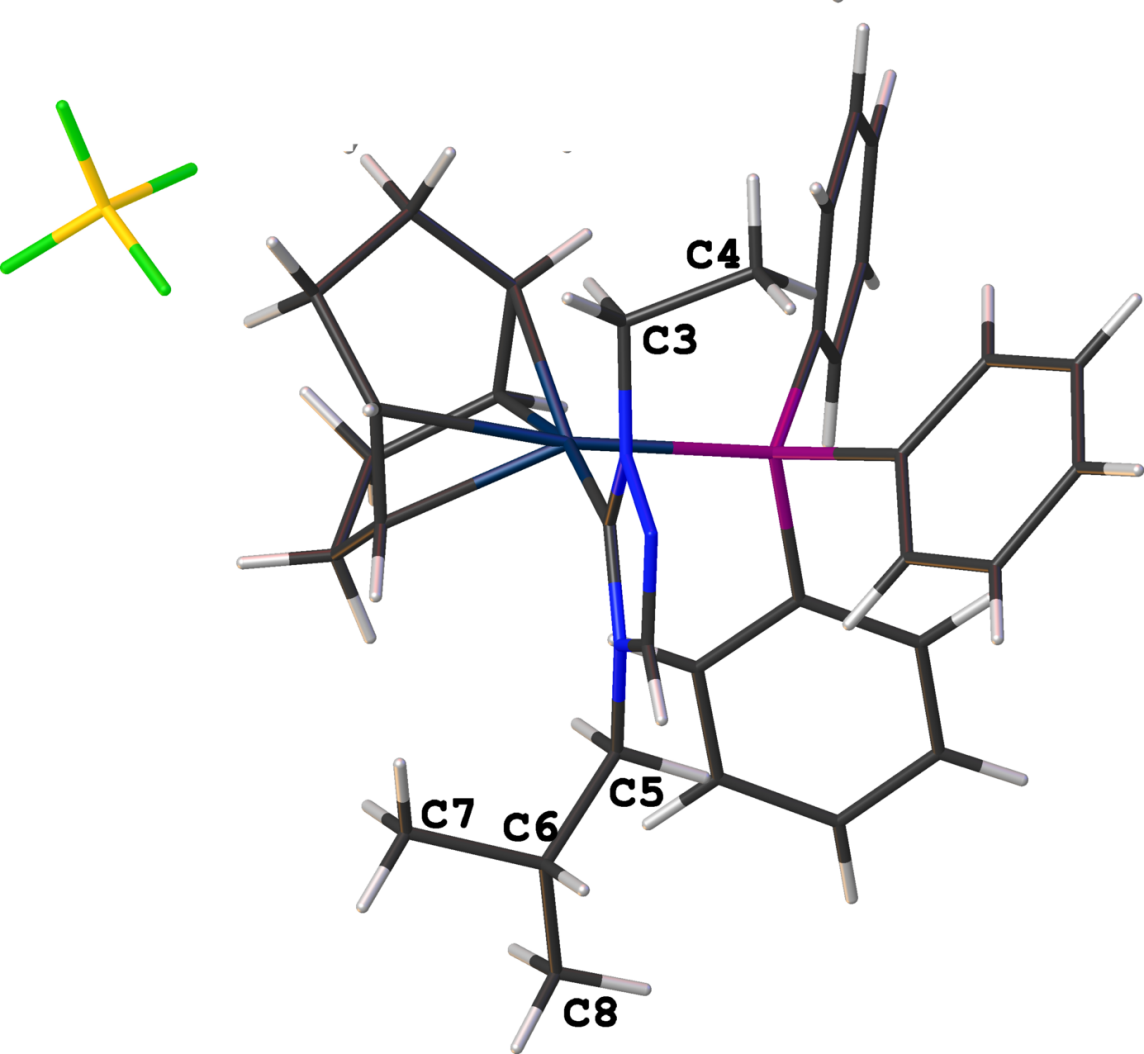
In the title compound (**3**), ethyl (C3 and C4) and isobutyl (C5–C7) wingtips exhibit an *anti* configuration with regards to the NHC.

**Figure 3:**
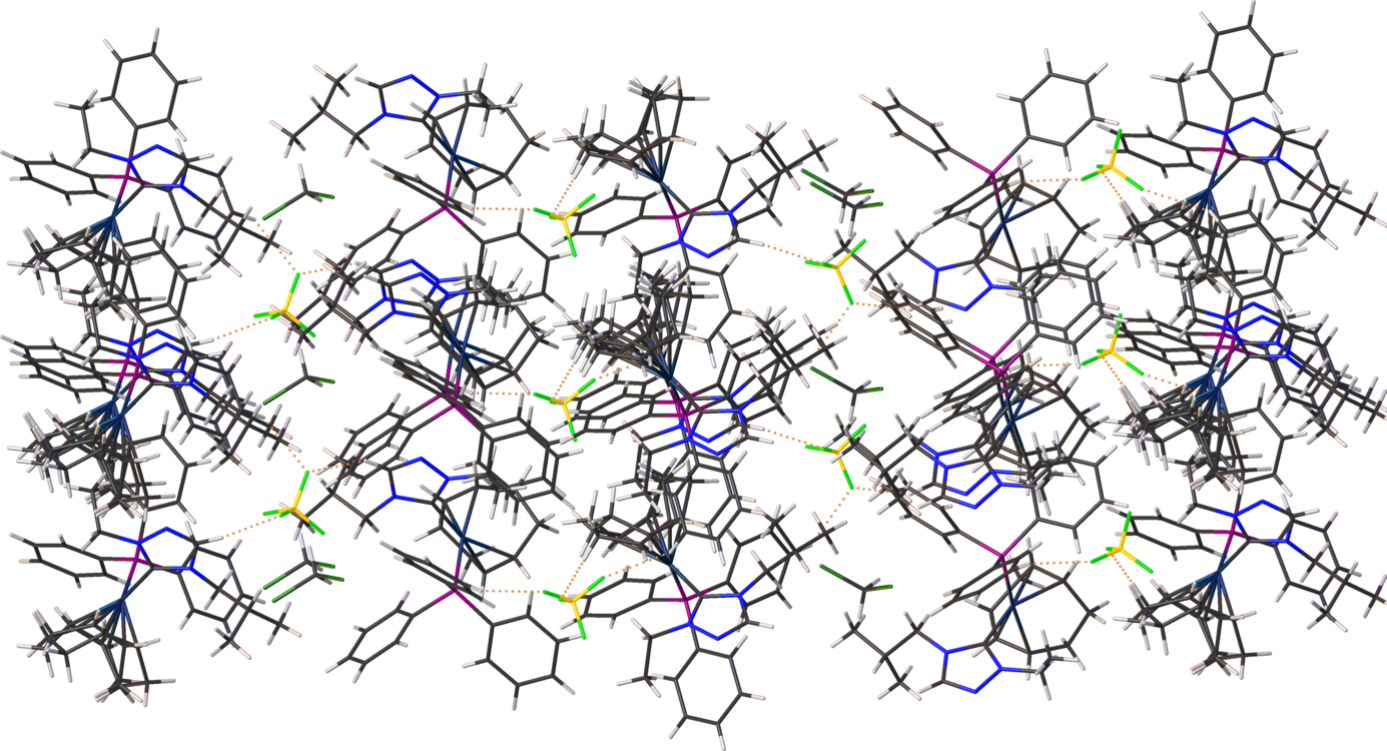
Mol­ecular packing diagram showing hydrogen-bonding inter­actions as dotted orange lines.

The coordination sphere around the Ir^I^ ion is formed by the bidentate (1,2,5,6-η)-cyclo­octa-1,5-diene (COD), NHC, and tri­phenyl­phosphane ligands, resulting in a distorted square-planar geometry. The distorted square-planar geometry around the Ir^I^ atoms is characterized by C_NHC_— Ir—P bond angles of 93.5 (5)° for cation A and 93.4 (4)° for cation B. The N—C—N bond angles of the NHC ligand are 103.9 (10) and 103.7 (10)° for cations *A* and *B*, respectively. Other selected bond lengths in cations *A* and *B* are Ir—C_NHC_ = 2.035 (13) and 2.029 (12) Å, and Ir—P = 2.323 (4) and 2.329 (4) Å. The wing tip substituents in the carbene ligand are *anti* with respect to one another as shown in Fig. 2 ▸. Fig. 3 ▸ shows several close F⋯H contacts (likely, non-standard hydrogen bonds) shown as dotted orange lines stabilizing the orientation of [BF_4_^−^], Table 1 ▸. Notably, the incorporated di­chloro­methane solvate does not exhibit hydrogen-bonding inter­actions with the cationic complex.

## Synthesis and crystallization

**Figure 4:**
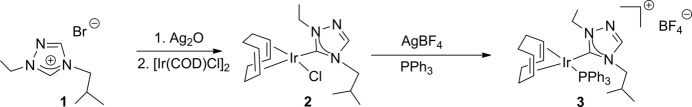
Reaction scheme for the synthesis of the title compound (**3**).

The synthesis of 1-ethyl-4-isobutyl-1,2,4-triazolium bromide (**1**) has been published previously (Lerch *et al.*, 2024*c* ▸). All other compounds used in the syntheses, shown in Fig. 4 ▸, were obtained from Sigma-Aldrich and Strem, and used as received; all syntheses were performed under a nitro­gen atmosphere. NMR spectra were recorded at room temperature in CDCl_3_ on a 400 MHz (operating at 100 MHz for ^13^C, and 162 MHz for ^31^P) Varian spectrometer and referenced to the residual solvent peak (δ in p.p.m.). The title compound (**3**) was crystallized by slow diffusion of pentane into a CH_2_Cl_2_ solution.

Chloro­[(1,2,5,6-η)-cyclo­octa-1,5-diene](1-ethyl-4-isobutyl-1,2,4-triazol-5-yl­idene)iridium(I) (**2**): Triazolium bromide (**1**) (0.070 g, 0.298 mmol) and Ag_2_O (0.035 g, 0.149 mmol) were stirred at room temperature in the dark for 1 h in CH_2_Cl_2_ (10 ml). The mixture was then filtered through Celite into [Ir(cod)Cl]_2_ (0.100 g, 0.149 mmol), and stirred again in the dark for 1.5 h. The resulting solution was filtered through Celite and the solvent was removed under reduced pressure in a rotavapor. The yellow–orange solid product (**2**) was dried under vacuum. Yield: 0.129 g (88.5%). ^1^H NMR: δ 7.83 (*s*, 1H, N—C3H—N), 4.73 (*q*, 2H, N—CH_2_ of eth­yl), 4.64 (*d*, 2H, N—CH_2_ of isobut­yl), 4.58 (*m*, 2H, CH of COD), 4.20 (*m*, 2H, CH of COD), 3.01, 2.84(*m*, 4H, CH_2_ of COD), 2.48, 2.27 (*m*, 4H, CH_2_ of COD), 1.88 (*m*, 1H, CH of isobut­yl), 1.54 (*t*, 3H, CH_3_ of eth­yl), 1.04 (*d*, 6H, CH_3_ of isobut­yl). ^13^C NMR: δ 182.54, 142.17 (N—C3H—N), 86.33, 85.66 (CH of COD), 55.94 (N—CH_2_ of isobut­yl), 47.78 (N—CH_2_ of eth­yl), 33.99, 33.00, 30.06, 29.03 (CH_2_ of COD), 28.91 (CH of isobut­yl), 20.28 (CH_3_ of isobut­yl), 15.36 (CH_3_ of eth­yl).

[(1,2,5,6-η)-Cyclo­octa-1,5-diene](1-ethyl-4-isobutyl-1,2,4-triazol-5-yl­idene)(tri­phenyl­phosphane)iridium(I) tetra­fluor­ido­borate (**3**): Tri­phenyl­phosphane (0.069 g, 0.264 mmol) and AgBF_4_ (0.051 g, 0.264 mmol) were added to (**2**) (0.129 g, 0.264 mmol) in CH_2_Cl_2_ (15 ml). The solution was stirred in the dark for 1.5 h. The resulting mixture was filtered through Celite and the solvent was removed under reduced pressure. The bright orange–red solid product (**3**) was dried under vacuum. Yield: 0.212 g (100%). ^1^H NMR: δ 8.08 (*s*, 1H, N—C3H—N), 7.48-7.25 (*m*, 15H, aromatic-H), 4.72 (*q*, 2H, N—CH_2_ of eth­yl), 4.47 (*d*, 2H, N—CH_2_ of isobut­yl), 4.24 (*m*, 2H, CH of COD), 4.08 (*m*, 2H, CH of COD), 2.60 (*m*, 4H, CH_2_ of COD), 2.48 (*m*, 2H, CH_2_ of COD), 2.23 (*m*, 2H, CH_2_ of COD), 2.06 (*m*, 1H, CH of isobut­yl), 1.23 (*t*, 3H, CH_3_ of eth­yl), 0.92 (*d*, 6H, CH_3_ of isobut­yl). ^13^C NMR: δ 178.03, 143.94 (N—C3H—N), 133.65–129.07 (C_aromatic_), 87.80, 87.69, 85.49, 85.37 (CH of COD), 55.66 (N—CH_2_ of isobut­yl), 47.90 (N—CH_2_ of eth­yl), 31.68, 31.67, 31.20, 31.16 (CH_2_ of COD), 28.09 (CH of isobut­yl), 19.95 (CH_3_ of isobut­yl), 13.83 (CH_3_ of eth­yl).31P NMR: δ 17.44.

## Refinement

Crystal data and refinement details are summarized in Table 2 ▸. The crystal grew as a non-merohedral twin. This was evident due to the double spots in the diffraction pattern and only a partial fit of all spots to the determined unit cell. Due to the large number of overlaps and almost pseudo-merohedral nature of the twinning, TWINROTMAT (Spek, 2020 ▸) was utilized to determine the twin matrix (−1 0 0 0 −1 0 0.146 0 1) after initial solution. Rotation frames were integrated using *CrysAlis PRO* (Rigaku OD, 2024 ▸), producing a listing of unaveraged *F*^2^ and σ(*F*^2^) values. All carbon atoms of the COD ligand of cation *B* are disordered over adjacent sites in a 0.50:0.50 ratio. The maximum and minimum electron density peaks of 1.91 and 1.42 e Å^−3^, respectively, are located 1.04 and 1.11 Å from the H32′ and H4′*C* atoms, respectively.

## Supplementary Material

Crystal structure: contains datablock(s) I. DOI: 10.1107/S2414314624009416/tk4109sup1.cif

Structure factors: contains datablock(s) I. DOI: 10.1107/S2414314624009416/tk4109Isup2.hkl

CCDC reference: 2386332

Additional supporting information:  crystallographic information; 3D view; checkCIF report

## Figures and Tables

**Table 1:** Hydrogen-bond geometry (Å, °)

*D*—H⋯*A*	*D*—H	H⋯*A*	*D*⋯*A*	*D*—H⋯*A*
C2—H2⋯F8^i^	0.95	2.32	3.18 (2)	149
C19—H19⋯F1^ii^	0.95	2.50	3.380 (19)	154
C2′—H2′⋯F5^iii^	0.95	2.26	3.171 (18)	161
C13′—H13′⋯F2^iii^	0.95	2.56	3.38 (2)	144
C18′—H18′⋯F8	0.95	2.52	3.319 (16)	142
C33"—H33*B*⋯F3^ii^	0.99	2.24	3.08 (3)	143

Symmetry codes: (i) 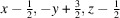; (ii) 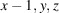; (iii) 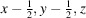.

**Table 2:** Experimental details

Crystal data	
Chemical formula	[Ir(C_8_H_12_)(C_8_H_15_N_3_)(C_18_H_15_P)]BF_4_·0.5CH_2_Cl_2_
*M*_r_	845.17
Crystal system, space group	Monoclinic, *Cc*
Temperature (K)	100
*a*, *b*, *c* (Å)	13.19118 (17), 13.5580 (2), 38.8216 (7)
β (°)	91.4236 (14)
*V* (Å^3^)	6940.96 (18)
*Z*	8
Radiation type	Mo *K*α
μ (mm^−1^)	4.02
Crystal size (mm)	0.17 × 0.11 × 0.02 × 0.81 (radius)

Data collection	
Diffractometer	Rigaku XtaLAB Synergy-S
Absorption correction	Multi-scan (*CrysAlis PRO*; Rigaku OD, 2024 ▸)
*T*_min_, *T*_max_	0.072, 0.097
No. of measured, independent and observed [*I* > 2σ(*I*)] reflections	71611, 16962, 16233
*R*_int_	0.056
(sin θ/λ)_max_ (Å^−1^)	0.667

Refinement	
*R*[*F*^2^ > 2σ(*F*^2^)], *wR*(*F*^2^), *S*	0.051, 0.133, 1.08
No. of reflections	16962
No. of parameters	889
No. of restraints	558
H-atom treatment	H-atom parameters constrained
Δρ_max_, Δρ_min_ (e Å^−3^)	1.91, −1.42
Absolute structure	Twinning involves inversion, so Flack parameter cannot be determined

Computer programs: *CrysAlis PRO* (Rigaku OD, 2024 ▸), *SHELXT* (Sheldrick, 2015*a* ▸), *SHELXL2018/3* (Sheldrick, 2015*b* ▸), *OLEX2* (Dolomanov *et al.*, 2009 ▸) and *publCIF* (Westrip, 2010 ▸).

## full crystallographic data

### [(1,2,5,6-η)-Cycloocta-1,5-diene](1-ethyl-4-isobutyl-1,2,4-triazol-5-ylidene)(triphenylphosphane)iridium(I) tetrafluoridoborate dichloromethane hemisolvate Crystal data

**Table:**

[Ir(C_8_H_12_)(C_8_H_15_N_3_)(C_18_H_15_P)]BF_4_·0.5CH_2_Cl_2_	*F*(000) = 3368
*M**_r_* = 845.17	*D*_x_ = 1.618 Mg m^−^^3^
Monoclinic, *C**c*	Mo *K*α radiation, λ = 0.71073 Å
*a* = 13.19118 (17) Å	Cell parameters from 42032 reflections
*b* = 13.5580 (2) Å	θ = 2.1–28.2°
*c* = 38.8216 (7) Å	µ = 4.02 mm^−^^1^
β = 91.4236 (14)°	*T* = 100 K
*V* = 6940.96 (18) Å^3^	Plate, red
*Z* = 8	0.17 × 0.11 × 0.02 × 0.81 (radius) mm

### [(1,2,5,6-η)-Cycloocta-1,5-diene](1-ethyl-4-isobutyl-1,2,4-triazol-5-ylidene)(triphenylphosphane)iridium(I) tetrafluoridoborate dichloromethane hemisolvate Data collection

**Table:**

Rigaku XtaLAB Synergy-S diffractometer	16233 reflections with *I* > 2σ(*I*)
Detector resolution: 10.0 pixels mm^-1^	*R*_int_ = 0.056
ω scans	θ_max_ = 28.3°, θ_min_ = 2.1°
Absorption correction: multi-scan (CrysAlisPro; Rigaku OD, 2024)	*h* = −16→17
*T*_min_ = 0.072, *T*_max_ = 0.097	*k* = −17→18
71611 measured reflections	*l* = −51→51
16962 independent reflections	

### [(1,2,5,6-η)-Cycloocta-1,5-diene](1-ethyl-4-isobutyl-1,2,4-triazol-5-ylidene)(triphenylphosphane)iridium(I) tetrafluoridoborate dichloromethane hemisolvate Refinement

**Table:**

Refinement on *F*^2^	Hydrogen site location: inferred from neighbouring sites
Least-squares matrix: full	H-atom parameters constrained
*R*[*F*^2^ > 2σ(*F*^2^)] = 0.051	*w* = 1/[σ^2^(*F*_o_^2^) + (0.0699*P*)^2^ + 71.6254*P*] where *P* = (*F*_o_^2^ + 2*F*_c_^2^)/3
*wR*(*F*^2^) = 0.133	(Δ/σ)_max_ < 0.001
*S* = 1.08	Δρ_max_ = 1.91 e Å^−^^3^
16962 reflections	Δρ_min_ = −1.42 e Å^−^^3^
889 parameters	Absolute structure: Twinning involves inversion, so Flack parameter cannot be determined
558 restraints	

### [(1,2,5,6-η)-Cycloocta-1,5-diene](1-ethyl-4-isobutyl-1,2,4-triazol-5-ylidene)(triphenylphosphane)iridium(I) tetrafluoridoborate dichloromethane hemisolvate Special details

**Table:**

Geometry. All esds (except the esd in the dihedral angle between two l.s. planes) are estimated using the full covariance matrix. The cell esds are taken into account individually in the estimation of esds in distances, angles and torsion angles; correlations between esds in cell parameters are only used when they are defined by crystal symmetry. An approximate (isotropic) treatment of cell esds is used for estimating esds involving l.s. planes.
Refinement. Refined as a 4-component inversion twin.

### [(1,2,5,6-η)-Cycloocta-1,5-diene](1-ethyl-4-isobutyl-1,2,4-triazol-5-ylidene)(triphenylphosphane)iridium(I) tetrafluoridoborate dichloromethane hemisolvate Fractional atomic coordinates and isotropic or equivalent isotropic displacement parameters (Å^2^)

**Table:**

	*x*	*y*	*z*	*U*_iso_*/*U*_eq_	Occ. (<1)
Ir1	0.12659 (3)	0.60645 (4)	0.29374 (2)	0.02226 (12)	
P1	0.0178 (3)	0.4738 (3)	0.28445 (10)	0.0200 (7)	
N1	−0.0280 (10)	0.7663 (8)	0.2806 (3)	0.025 (2)	
N2	−0.0786 (11)	0.8231 (10)	0.2561 (3)	0.033 (3)	
N3	0.0287 (10)	0.7215 (9)	0.2317 (3)	0.027 (3)	
C1	0.0370 (11)	0.7016 (10)	0.2662 (3)	0.026 (3)	
C2	−0.0430 (13)	0.7942 (11)	0.2273 (4)	0.036 (4)	
H2	−0.063878	0.820010	0.205533	0.043*	
C3	−0.0482 (11)	0.7760 (11)	0.3170 (3)	0.029 (3)	
H3A	−0.043268	0.846280	0.323848	0.035*	
H3B	0.003272	0.738623	0.330679	0.035*	
C4	−0.1546 (13)	0.7367 (14)	0.3248 (5)	0.046 (5)	
H4A	−0.205463	0.774253	0.311413	0.068*	
H4B	−0.167214	0.744030	0.349407	0.068*	
H4C	−0.158926	0.666890	0.318403	0.068*	
C5	0.0804 (15)	0.6657 (12)	0.2052 (4)	0.039 (4)	
H5A	0.032635	0.616715	0.195117	0.047*	
H5B	0.137623	0.629090	0.216148	0.047*	
C6	0.1211 (15)	0.7302 (13)	0.1763 (4)	0.049 (5)	
H6	0.063574	0.768541	0.165669	0.059*	
C7	0.2020 (17)	0.8026 (18)	0.1900 (6)	0.060 (6)	
H7A	0.172294	0.846075	0.207149	0.090*	
H7B	0.226990	0.842240	0.170837	0.090*	
H7C	0.258462	0.765626	0.200583	0.090*	
C8	0.166 (2)	0.6631 (16)	0.1488 (6)	0.067 (8)	
H8A	0.225101	0.628311	0.158588	0.100*	
H8B	0.186795	0.703188	0.129176	0.100*	
H8C	0.114887	0.615061	0.141036	0.100*	
C9	0.0541 (9)	0.4071 (9)	0.2458 (3)	0.021 (3)	
C10	0.1512 (11)	0.4195 (11)	0.2325 (4)	0.033 (3)	
H10	0.197890	0.463963	0.243160	0.040*	
C11	0.1784 (13)	0.3665 (13)	0.2036 (4)	0.037 (4)	
H11	0.244340	0.374900	0.194700	0.045*	
C12	0.1115 (12)	0.3014 (13)	0.1873 (4)	0.041 (4)	
H12	0.130642	0.266107	0.167400	0.049*	
C13	0.0159 (12)	0.2891 (13)	0.2009 (4)	0.040 (4)	
H13	−0.029812	0.243165	0.190576	0.048*	
C14	−0.0142 (12)	0.3431 (12)	0.2296 (4)	0.033 (4)	
H14	−0.080860	0.336111	0.237949	0.040*	
C15	−0.1178 (10)	0.4993 (10)	0.2758 (3)	0.021 (3)	
C16	−0.1438 (10)	0.5503 (10)	0.2457 (3)	0.024 (3)	
H16	−0.091624	0.567276	0.230348	0.029*	
C17	−0.2428 (10)	0.5776 (12)	0.2372 (4)	0.034 (4)	
H17	−0.258423	0.611738	0.216376	0.041*	
C18	−0.3187 (11)	0.5533 (12)	0.2603 (4)	0.034 (4)	
H18	−0.386644	0.573259	0.255525	0.040*	
C19	−0.2954 (10)	0.5002 (12)	0.2900 (4)	0.031 (3)	
H19	−0.347643	0.482538	0.305264	0.037*	
C20	−0.1948 (8)	0.4726 (11)	0.2976 (4)	0.024 (3)	
H20	−0.179276	0.435424	0.317807	0.029*	
C21	0.0219 (11)	0.3790 (9)	0.3183 (3)	0.024 (2)	
C22	−0.0174 (12)	0.4027 (10)	0.3503 (3)	0.026 (2)	
H22	−0.048227	0.465012	0.353977	0.032*	
C23	−0.0107 (11)	0.3333 (9)	0.3770 (4)	0.029 (2)	
H23	−0.037786	0.348646	0.398802	0.035*	
C24	0.0347 (11)	0.2427 (10)	0.3720 (4)	0.029 (2)	
H24	0.039152	0.196453	0.390379	0.035*	
C25	0.0740 (11)	0.2193 (10)	0.3402 (3)	0.027 (2)	
H25	0.105186	0.157088	0.336635	0.032*	
C26	0.0673 (11)	0.2875 (8)	0.3135 (4)	0.024 (2)	
H26	0.094157	0.271452	0.291731	0.029*	
C27	0.2489 (11)	0.5091 (12)	0.3141 (4)	0.031 (3)	
H27	0.236442	0.437273	0.310055	0.037*	
C28	0.1966 (12)	0.5479 (10)	0.3413 (4)	0.031 (2)	
H28	0.153493	0.498441	0.353200	0.037*	
C29	0.2301 (13)	0.6338 (10)	0.3643 (4)	0.035 (3)	
H29E	0.280905	0.609749	0.381471	0.041*	
H29F	0.170805	0.658096	0.376937	0.041*	
C30	0.2759 (13)	0.7200 (12)	0.3442 (4)	0.036 (3)	
H30E	0.348691	0.706864	0.340667	0.043*	
H30F	0.270654	0.781127	0.358030	0.043*	
C31	0.2222 (12)	0.7357 (13)	0.3090 (4)	0.038 (3)	
H31	0.187367	0.800936	0.306441	0.045*	
C32	0.2560 (12)	0.6968 (12)	0.2788 (4)	0.037 (3)	
H32	0.242738	0.739168	0.258104	0.045*	
C33	0.3472 (13)	0.6270 (12)	0.2757 (5)	0.040 (3)	
H33E	0.344202	0.596074	0.252558	0.048*	
H33F	0.410206	0.666569	0.277279	0.048*	
C34	0.3535 (11)	0.5449 (11)	0.3029 (4)	0.036 (3)	
H34E	0.391385	0.488371	0.293545	0.043*	
H34F	0.391836	0.569651	0.323416	0.043*	
Ir1'	−0.01715 (3)	0.52789 (4)	0.48110 (2)	0.02538 (13)	
P1'	0.1221 (3)	0.4294 (3)	0.49566 (10)	0.0213 (7)	
N1'	−0.1804 (9)	0.3761 (9)	0.4955 (3)	0.027 (2)	
N2'	−0.2398 (10)	0.3379 (10)	0.5211 (3)	0.030 (2)	
N3'	−0.1352 (9)	0.4473 (10)	0.5432 (3)	0.027 (2)	
C1'	−0.1142 (10)	0.4454 (10)	0.5088 (3)	0.026 (2)	
C2'	−0.2114 (12)	0.3794 (12)	0.5492 (4)	0.030 (2)	
H2'	−0.238496	0.365732	0.571106	0.037*	
C3'	−0.1921 (13)	0.3438 (10)	0.4603 (3)	0.030 (3)	
H3'A	−0.154780	0.389443	0.445232	0.036*	
H3'B	−0.264699	0.346570	0.453304	0.036*	
C4'	−0.1526 (13)	0.2384 (10)	0.4550 (4)	0.034 (4)	
H4'A	−0.152985	0.223221	0.430292	0.051*	
H4'B	−0.196430	0.191661	0.466804	0.051*	
H4'C	−0.083257	0.233195	0.464439	0.051*	
C5'	−0.0792 (11)	0.5054 (13)	0.5686 (4)	0.032 (3)	
H5'A	−0.038642	0.555407	0.556500	0.038*	
H5'B	−0.031256	0.461458	0.581277	0.038*	
C6'	−0.1455 (13)	0.5580 (14)	0.5948 (4)	0.044 (4)	
H6'	−0.190953	0.508128	0.605391	0.052*	
C7'	−0.2117 (18)	0.6360 (17)	0.5766 (6)	0.064 (7)	
H7'A	−0.170537	0.673526	0.560546	0.097*	
H7'B	−0.239346	0.680841	0.593824	0.097*	
H7'C	−0.267439	0.603502	0.563944	0.097*	
C8'	−0.0759 (17)	0.601 (2)	0.6231 (6)	0.061 (6)	
H8'A	−0.033369	0.548473	0.633063	0.092*	
H8'B	−0.117160	0.630035	0.641178	0.092*	
H8'C	−0.032677	0.652074	0.613316	0.092*	
C9'	0.0980 (11)	0.3001 (9)	0.5057 (3)	0.027 (2)	
C10'	0.0410 (11)	0.2791 (9)	0.5344 (4)	0.028 (2)	
H10'	0.020521	0.331859	0.548732	0.033*	
C11'	0.0132 (12)	0.1838 (9)	0.5428 (4)	0.031 (2)	
H11'	−0.022872	0.171862	0.563307	0.037*	
C12'	0.0377 (11)	0.1063 (10)	0.5216 (4)	0.031 (2)	
H12'	0.014961	0.041275	0.526352	0.037*	
C13'	0.0965 (12)	0.1254 (10)	0.4930 (4)	0.031 (2)	
H13'	0.117328	0.072206	0.478898	0.037*	
C14'	0.1252 (11)	0.2211 (8)	0.4847 (4)	0.030 (2)	
H14'	0.163557	0.232828	0.464712	0.036*	
C15'	0.1889 (8)	0.4708 (7)	0.5348 (2)	0.030 (2)	
C16'	0.1769 (8)	0.5668 (7)	0.5466 (3)	0.033 (3)	
H16'	0.131411	0.610397	0.534945	0.040*	
C17'	0.2313 (9)	0.5988 (6)	0.5756 (3)	0.038 (3)	
H17'	0.223110	0.664352	0.583759	0.046*	
C18'	0.2978 (9)	0.5349 (9)	0.5928 (3)	0.043 (3)	
H18'	0.335059	0.556851	0.612587	0.052*	
C19'	0.3099 (9)	0.4390 (8)	0.5809 (3)	0.042 (3)	
H19'	0.355311	0.395393	0.592602	0.050*	
C20'	0.2554 (9)	0.4070 (6)	0.5519 (3)	0.036 (3)	
H20'	0.263613	0.341436	0.543788	0.043*	
C21'	0.2197 (9)	0.4283 (10)	0.4638 (3)	0.023 (2)	
C22'	0.3140 (9)	0.4729 (11)	0.4687 (4)	0.026 (2)	
H22'	0.331499	0.501210	0.490445	0.032*	
C23'	0.3829 (11)	0.4764 (12)	0.4422 (3)	0.032 (2)	
H23'	0.446755	0.507547	0.445894	0.038*	
C24'	0.3587 (11)	0.4348 (11)	0.4105 (4)	0.031 (2)	
H24'	0.406335	0.436642	0.392515	0.037*	
C25'	0.2650 (10)	0.3904 (11)	0.4048 (4)	0.030 (2)	
H25'	0.248114	0.360703	0.383286	0.037*	
C26'	0.1964 (11)	0.3904 (11)	0.4313 (3)	0.027 (2)	
H26'	0.130800	0.363305	0.427109	0.033*	
C27"	0.044 (3)	0.593 (3)	0.4333 (10)	0.026 (3)	0.5
H27"	0.094819	0.551053	0.421195	0.031*	0.5
C27'	0.058 (3)	0.604 (3)	0.4385 (9)	0.026 (3)	0.5
H27'	0.116910	0.566659	0.429058	0.031*	0.5
C28"	0.082 (3)	0.642 (3)	0.4619 (10)	0.026 (3)	0.5
H28"	0.155691	0.631468	0.467104	0.032*	0.5
C28'	0.083 (3)	0.658 (3)	0.4676 (10)	0.026 (3)	0.5
H28'	0.154870	0.650793	0.475742	0.031*	0.5
C29"	0.039 (2)	0.740 (3)	0.4755 (13)	0.028 (3)	0.5
H29A	0.065259	0.794456	0.461125	0.033*	0.5
H29B	0.066450	0.750435	0.499266	0.033*	0.5
C29'	0.033 (3)	0.756 (3)	0.4776 (12)	0.028 (3)	0.5
H29C	0.084566	0.798620	0.489027	0.034*	0.5
H29D	0.009357	0.790267	0.456242	0.034*	0.5
C30"	−0.0776 (19)	0.7476 (18)	0.4760 (9)	0.027 (3)	0.5
H30A	−0.097894	0.791605	0.494999	0.033*	0.5
H30B	−0.103419	0.776278	0.454033	0.033*	0.5
C30'	−0.058 (2)	0.7451 (18)	0.5016 (8)	0.028 (3)	0.5
H30C	−0.033225	0.743225	0.525897	0.033*	0.5
H30D	−0.103938	0.802277	0.498748	0.033*	0.5
C31"	−0.124 (2)	0.6450 (18)	0.4810 (8)	0.025 (3)	0.5
H31"	−0.170445	0.641916	0.500841	0.030*	0.5
C31'	−0.116 (3)	0.649 (2)	0.4928 (8)	0.026 (3)	0.5
H31'	−0.162071	0.631098	0.511784	0.031*	0.5
C32"	−0.155 (3)	0.588 (2)	0.4535 (6)	0.026 (3)	0.5
H32"	−0.218744	0.549300	0.457543	0.031*	0.5
C32'	−0.150 (3)	0.611 (2)	0.4618 (6)	0.026 (3)	0.5
H32'	−0.216670	0.575259	0.462433	0.031*	0.5
C33"	−0.1362 (18)	0.611 (2)	0.4155 (7)	0.026 (3)	0.5
H33A	−0.186797	0.659426	0.407114	0.031*	0.5
H33B	−0.145913	0.549543	0.401763	0.031*	0.5
C33'	−0.127 (2)	0.661 (2)	0.4274 (7)	0.027 (3)	0.5
H33C	−0.183783	0.650139	0.410755	0.033*	0.5
H33D	−0.120251	0.733366	0.431049	0.033*	0.5
C34"	−0.0288 (19)	0.651 (2)	0.4095 (9)	0.027 (3)	0.5
H34A	−0.010514	0.642505	0.385159	0.032*	0.5
H34B	−0.025872	0.722383	0.415180	0.032*	0.5
C34'	−0.0283 (19)	0.622 (2)	0.4120 (8)	0.027 (3)	0.5
H34C	−0.043514	0.558716	0.400071	0.032*	0.5
H34D	−0.004811	0.669095	0.394547	0.032*	0.5
Cl1	0.5771 (7)	0.5278 (8)	0.6159 (3)	0.102 (3)	
Cl2	0.4852 (9)	0.4734 (6)	0.6780 (3)	0.106 (3)	
C35	0.586 (3)	0.462 (3)	0.6547 (11)	0.106 (6)	
H35A	0.597467	0.391625	0.649642	0.127*	
H35B	0.645995	0.486389	0.668252	0.127*	
F1	0.5708 (10)	0.4784 (10)	0.3628 (3)	0.053 (3)	
F2	0.6253 (10)	0.5096 (16)	0.4169 (4)	0.088 (6)	
F3	0.7346 (10)	0.4513 (11)	0.3786 (4)	0.062 (4)	
F4	0.6099 (13)	0.3549 (12)	0.3985 (4)	0.085 (6)	
B1	0.635 (2)	0.450 (2)	0.3893 (6)	0.052 (7)	
F5	0.2490 (9)	0.8110 (9)	0.6258 (3)	0.046 (3)	
F6	0.4174 (9)	0.8114 (8)	0.6123 (3)	0.046 (3)	
F7	0.3683 (15)	0.8643 (12)	0.6646 (4)	0.087 (6)	
F8	0.3589 (11)	0.7035 (11)	0.6517 (3)	0.057 (3)	
B2	0.348 (2)	0.7976 (18)	0.6386 (6)	0.051 (6)	

### [(1,2,5,6-η)-Cycloocta-1,5-diene](1-ethyl-4-isobutyl-1,2,4-triazol-5-ylidene)(triphenylphosphane)iridium(I) tetrafluoridoborate dichloromethane hemisolvate Atomic displacement parameters (Å^2^)

**Table:**

	*U*^11^	*U*^22^	*U*^33^	*U*^12^	*U*^13^	*U*^23^
Ir1	0.0198 (2)	0.0156 (2)	0.0313 (2)	−0.0022 (2)	−0.00146 (19)	0.0036 (2)
P1	0.0198 (18)	0.0155 (16)	0.0247 (17)	−0.0010 (13)	0.0018 (13)	0.0004 (13)
N1	0.033 (7)	0.016 (6)	0.026 (6)	0.001 (5)	0.000 (5)	−0.003 (4)
N2	0.042 (8)	0.023 (6)	0.034 (7)	0.012 (6)	−0.005 (6)	−0.003 (5)
N3	0.029 (7)	0.025 (6)	0.028 (6)	0.005 (5)	0.002 (5)	0.006 (5)
C1	0.025 (7)	0.023 (7)	0.030 (7)	0.001 (6)	0.003 (6)	−0.001 (6)
C2	0.043 (10)	0.026 (8)	0.039 (9)	0.006 (7)	−0.001 (7)	0.011 (6)
C3	0.039 (9)	0.021 (7)	0.028 (7)	0.007 (6)	−0.003 (6)	−0.004 (5)
C4	0.055 (12)	0.038 (10)	0.045 (11)	0.013 (9)	0.028 (9)	0.006 (8)
C5	0.044 (10)	0.042 (10)	0.033 (8)	−0.006 (8)	0.012 (7)	0.003 (7)
C6	0.056 (12)	0.051 (11)	0.042 (10)	0.008 (10)	0.010 (9)	0.017 (9)
C7	0.055 (14)	0.062 (15)	0.063 (14)	−0.009 (12)	0.012 (11)	0.019 (11)
C8	0.091 (19)	0.053 (14)	0.059 (14)	0.002 (13)	0.043 (14)	0.019 (11)
C9	0.027 (7)	0.016 (6)	0.021 (6)	0.004 (5)	0.003 (5)	0.004 (5)
C10	0.027 (8)	0.031 (8)	0.042 (9)	0.007 (6)	0.006 (7)	0.007 (6)
C11	0.030 (9)	0.039 (10)	0.043 (10)	0.012 (8)	0.010 (7)	0.000 (8)
C12	0.048 (11)	0.046 (10)	0.029 (8)	0.019 (9)	0.006 (8)	−0.006 (7)
C13	0.048 (11)	0.030 (8)	0.042 (10)	0.007 (8)	−0.014 (8)	−0.012 (7)
C14	0.032 (9)	0.036 (9)	0.033 (8)	−0.001 (7)	−0.001 (6)	−0.005 (6)
C15	0.027 (7)	0.014 (6)	0.023 (7)	−0.007 (5)	−0.005 (5)	−0.003 (5)
C16	0.029 (8)	0.017 (6)	0.028 (7)	0.001 (5)	0.005 (6)	0.005 (5)
C17	0.036 (9)	0.031 (8)	0.036 (9)	−0.004 (7)	0.001 (7)	0.007 (7)
C18	0.023 (8)	0.032 (8)	0.046 (9)	−0.002 (6)	−0.006 (7)	0.000 (7)
C19	0.014 (6)	0.035 (8)	0.043 (9)	0.003 (6)	0.003 (6)	0.004 (7)
C20	0.011 (6)	0.030 (7)	0.031 (7)	−0.007 (5)	−0.001 (5)	0.001 (6)
C21	0.026 (5)	0.015 (4)	0.029 (5)	−0.005 (4)	−0.004 (4)	−0.003 (4)
C22	0.026 (5)	0.022 (5)	0.031 (5)	−0.003 (4)	0.002 (4)	−0.001 (4)
C23	0.026 (5)	0.029 (5)	0.031 (5)	−0.005 (4)	0.000 (4)	0.001 (4)
C24	0.025 (5)	0.028 (5)	0.034 (5)	−0.005 (4)	−0.005 (4)	0.005 (4)
C25	0.025 (5)	0.022 (5)	0.034 (5)	−0.003 (4)	−0.003 (4)	0.006 (4)
C26	0.024 (5)	0.017 (5)	0.032 (5)	−0.004 (4)	0.001 (4)	0.000 (4)
C27	0.027 (5)	0.020 (5)	0.045 (6)	−0.004 (4)	−0.005 (5)	0.000 (5)
C28	0.027 (5)	0.020 (5)	0.044 (6)	−0.008 (4)	−0.006 (5)	0.001 (5)
C29	0.029 (5)	0.028 (5)	0.046 (6)	−0.006 (5)	−0.005 (5)	−0.002 (5)
C30	0.026 (5)	0.030 (5)	0.052 (6)	−0.005 (5)	−0.006 (5)	0.002 (5)
C31	0.029 (5)	0.028 (5)	0.056 (6)	−0.009 (5)	−0.009 (5)	0.005 (5)
C32	0.025 (5)	0.033 (6)	0.053 (6)	−0.008 (5)	−0.005 (5)	0.009 (5)
C33	0.033 (5)	0.033 (6)	0.055 (6)	−0.004 (5)	−0.001 (5)	0.003 (5)
C34	0.033 (5)	0.024 (5)	0.050 (6)	−0.002 (5)	0.001 (5)	0.000 (5)
Ir1'	0.0153 (2)	0.0190 (2)	0.0422 (3)	0.0032 (2)	0.0069 (2)	0.0088 (2)
P1'	0.0140 (17)	0.0199 (17)	0.0301 (18)	0.0021 (14)	0.0028 (13)	0.0029 (14)
N1'	0.022 (4)	0.025 (5)	0.036 (5)	−0.007 (4)	0.008 (4)	0.006 (4)
N2'	0.024 (5)	0.032 (5)	0.036 (5)	−0.007 (4)	0.006 (4)	0.008 (4)
N3'	0.020 (4)	0.028 (5)	0.034 (5)	0.000 (4)	0.004 (4)	−0.001 (4)
C1'	0.020 (5)	0.024 (5)	0.033 (5)	0.001 (4)	0.009 (4)	0.002 (4)
C2'	0.023 (5)	0.033 (5)	0.036 (5)	−0.002 (4)	0.005 (4)	0.006 (4)
C3'	0.034 (9)	0.023 (7)	0.033 (8)	0.004 (7)	0.000 (7)	0.009 (6)
C4'	0.038 (9)	0.029 (8)	0.034 (8)	−0.007 (7)	−0.004 (7)	0.001 (7)
C5'	0.026 (8)	0.036 (9)	0.032 (8)	0.000 (7)	−0.004 (6)	−0.005 (7)
C6'	0.038 (10)	0.043 (11)	0.049 (11)	0.011 (8)	0.008 (8)	−0.006 (8)
C7'	0.048 (13)	0.064 (15)	0.081 (17)	0.029 (12)	−0.006 (12)	−0.020 (12)
C8'	0.050 (13)	0.066 (16)	0.068 (15)	0.008 (12)	0.002 (11)	−0.010 (12)
C9'	0.019 (5)	0.017 (5)	0.045 (5)	0.000 (4)	−0.001 (4)	0.005 (4)
C10'	0.022 (5)	0.016 (5)	0.044 (6)	0.003 (4)	0.001 (4)	0.004 (4)
C11'	0.023 (5)	0.022 (5)	0.049 (6)	0.002 (4)	0.006 (5)	0.008 (5)
C12'	0.024 (5)	0.018 (5)	0.051 (6)	0.000 (4)	0.003 (5)	0.008 (5)
C13'	0.024 (5)	0.018 (5)	0.052 (6)	0.004 (4)	0.002 (5)	0.002 (4)
C14'	0.021 (5)	0.019 (5)	0.050 (6)	−0.004 (4)	0.000 (5)	0.000 (4)
C15'	0.031 (5)	0.029 (5)	0.031 (5)	0.000 (5)	0.004 (5)	0.002 (4)
C16'	0.034 (5)	0.029 (5)	0.037 (5)	0.000 (5)	0.003 (5)	0.001 (5)
C17'	0.040 (6)	0.036 (6)	0.039 (6)	−0.004 (5)	0.002 (5)	−0.003 (5)
C18'	0.046 (6)	0.046 (6)	0.038 (6)	0.000 (5)	0.001 (5)	−0.009 (5)
C19'	0.044 (6)	0.043 (6)	0.038 (6)	0.001 (5)	0.000 (5)	0.001 (5)
C20'	0.042 (6)	0.034 (6)	0.032 (5)	0.000 (5)	−0.001 (5)	0.004 (5)
C21'	0.019 (5)	0.017 (4)	0.034 (5)	0.009 (4)	−0.003 (4)	−0.001 (4)
C22'	0.020 (5)	0.024 (5)	0.035 (5)	0.006 (4)	0.003 (4)	0.006 (4)
C23'	0.025 (5)	0.029 (5)	0.041 (5)	0.007 (4)	0.006 (5)	0.007 (5)
C24'	0.029 (5)	0.026 (5)	0.037 (5)	0.012 (4)	0.005 (5)	0.007 (5)
C25'	0.032 (5)	0.025 (5)	0.035 (5)	0.010 (4)	0.001 (4)	0.004 (4)
C26'	0.028 (5)	0.021 (5)	0.033 (5)	0.010 (4)	−0.004 (4)	0.002 (4)
C27"	0.022 (5)	0.017 (6)	0.040 (6)	0.005 (5)	0.003 (4)	0.006 (5)
C27'	0.022 (5)	0.017 (6)	0.040 (6)	0.005 (5)	0.003 (5)	0.006 (5)
C28"	0.022 (5)	0.017 (6)	0.040 (6)	0.003 (5)	0.003 (5)	0.006 (5)
C28'	0.022 (5)	0.016 (6)	0.039 (6)	0.004 (5)	0.002 (5)	0.005 (5)
C29"	0.023 (4)	0.018 (6)	0.041 (6)	0.004 (5)	0.000 (5)	0.004 (5)
C29'	0.024 (4)	0.020 (6)	0.041 (6)	0.003 (5)	0.000 (5)	0.003 (5)
C30"	0.021 (5)	0.020 (5)	0.041 (6)	0.003 (5)	−0.001 (5)	0.003 (5)
C30'	0.022 (5)	0.020 (5)	0.041 (6)	0.004 (4)	0.000 (5)	0.004 (5)
C31"	0.018 (5)	0.017 (5)	0.041 (6)	0.006 (4)	0.001 (5)	0.004 (5)
C31'	0.019 (5)	0.017 (5)	0.042 (6)	0.004 (4)	0.001 (5)	0.003 (5)
C32"	0.019 (5)	0.017 (6)	0.041 (6)	0.006 (5)	0.001 (5)	0.004 (5)
C32'	0.019 (5)	0.017 (6)	0.041 (6)	0.006 (5)	0.001 (5)	0.004 (5)
C33"	0.021 (5)	0.017 (6)	0.040 (6)	0.007 (5)	0.003 (5)	0.005 (5)
C33'	0.022 (5)	0.018 (6)	0.042 (6)	0.005 (5)	0.001 (5)	0.005 (5)
C34"	0.022 (5)	0.018 (6)	0.040 (6)	0.005 (5)	0.003 (5)	0.006 (5)
C34'	0.023 (5)	0.017 (6)	0.041 (6)	0.006 (5)	0.003 (4)	0.005 (5)
Cl1	0.076 (5)	0.133 (8)	0.097 (6)	0.013 (5)	0.004 (4)	0.030 (5)
Cl2	0.152 (8)	0.063 (4)	0.104 (6)	0.023 (5)	0.019 (6)	0.012 (4)
C35	0.111 (11)	0.095 (10)	0.110 (10)	0.015 (10)	0.004 (10)	0.026 (10)
F1	0.064 (8)	0.057 (8)	0.040 (6)	0.002 (6)	0.003 (5)	0.021 (5)
F2	0.031 (7)	0.159 (17)	0.073 (9)	0.009 (9)	0.010 (6)	−0.052 (10)
F3	0.057 (8)	0.062 (8)	0.070 (9)	0.015 (6)	0.044 (7)	0.005 (7)
F4	0.078 (10)	0.071 (10)	0.108 (12)	0.031 (8)	0.041 (9)	0.064 (9)
B1	0.055 (14)	0.064 (15)	0.039 (11)	0.035 (13)	0.017 (10)	0.020 (10)
F5	0.049 (7)	0.051 (7)	0.039 (6)	−0.012 (6)	0.012 (5)	0.001 (5)
F6	0.053 (7)	0.040 (6)	0.045 (6)	−0.009 (5)	0.011 (5)	0.005 (5)
F7	0.128 (15)	0.065 (10)	0.069 (9)	−0.057 (10)	0.009 (9)	−0.023 (8)
F8	0.078 (9)	0.062 (8)	0.032 (6)	−0.009 (7)	0.004 (6)	0.002 (5)
B2	0.087 (19)	0.039 (12)	0.027 (10)	−0.022 (12)	0.014 (11)	0.007 (8)

### [(1,2,5,6-η)-Cycloocta-1,5-diene](1-ethyl-4-isobutyl-1,2,4-triazol-5-ylidene)(triphenylphosphane)iridium(I) tetrafluoridoborate dichloromethane hemisolvate Geometric parameters (Å, º)

**Table:**

Ir1—P1	2.323 (4)	N3'—C2'	1.387 (19)
Ir1—C1	2.035 (13)	N3'—C5'	1.451 (12)
Ir1—C27	2.215 (17)	C2'—H2'	0.9500
Ir1—C28	2.192 (16)	C3'—H3'A	0.9900
Ir1—C31	2.231 (17)	C3'—H3'B	0.9900
Ir1—C32	2.191 (17)	C3'—C4'	1.536 (12)
P1—C9	1.828 (13)	C4'—H4'A	0.9800
P1—C15	1.845 (14)	C4'—H4'B	0.9800
P1—C21	1.839 (14)	C4'—H4'C	0.9800
N1—N2	1.384 (11)	C5'—H5'A	0.9900
N1—C1	1.357 (12)	C5'—H5'B	0.9900
N1—C3	1.450 (11)	C5'—C6'	1.533 (13)
N2—C2	1.284 (12)	C6'—H6'	1.0000
N3—C1	1.370 (12)	C6'—C7'	1.531 (13)
N3—C2	1.373 (12)	C6'—C8'	1.531 (13)
N3—C5	1.459 (12)	C7'—H7'A	0.9800
C2—H2	0.9500	C7'—H7'B	0.9800
C3—H3A	0.9900	C7'—H7'C	0.9800
C3—H3B	0.9900	C8'—H8'A	0.9800
C3—C4	1.538 (13)	C8'—H8'B	0.9800
C4—H4A	0.9800	C8'—H8'C	0.9800
C4—H4B	0.9800	C9'—C10'	1.390 (12)
C4—H4C	0.9800	C9'—C14'	1.397 (12)
C5—H5A	0.9900	C10'—H10'	0.9500
C5—H5B	0.9900	C10'—C11'	1.384 (12)
C5—C6	1.531 (13)	C11'—H11'	0.9500
C6—H6	1.0000	C11'—C12'	1.380 (12)
C6—C7	1.535 (14)	C12'—H12'	0.9500
C6—C8	1.533 (13)	C12'—C13'	1.396 (12)
C7—H7A	0.9800	C13'—H13'	0.9500
C7—H7B	0.9800	C13'—C14'	1.392 (12)
C7—H7C	0.9800	C14'—H14'	0.9500
C8—H8A	0.9800	C15'—C16'	1.3900
C8—H8B	0.9800	C15'—C20'	1.3900
C8—H8C	0.9800	C16'—H16'	0.9500
C9—C10	1.402 (12)	C16'—C17'	1.3900
C9—C14	1.389 (12)	C17'—H17'	0.9500
C10—H10	0.9500	C17'—C18'	1.3900
C10—C11	1.387 (12)	C18'—H18'	0.9500
C11—H11	0.9500	C18'—C19'	1.3900
C11—C12	1.388 (13)	C19'—H19'	0.9500
C12—H12	0.9500	C19'—C20'	1.3900
C12—C13	1.389 (13)	C20'—H20'	0.9500
C13—H13	0.9500	C21'—C22'	1.391 (12)
C13—C14	1.397 (12)	C21'—C26'	1.390 (12)
C14—H14	0.9500	C22'—H22'	0.9500
C15—C16	1.392 (12)	C22'—C23'	1.390 (12)
C15—C20	1.387 (12)	C23'—H23'	0.9500
C16—H16	0.9500	C23'—C24'	1.386 (13)
C16—C17	1.388 (12)	C24'—H24'	0.9500
C17—H17	0.9500	C24'—C25'	1.387 (12)
C17—C18	1.399 (12)	C25'—H25'	0.9500
C18—H18	0.9500	C25'—C26'	1.387 (12)
C18—C19	1.390 (12)	C26'—H26'	0.9500
C19—H19	0.9500	C27"—H27"	1.0000
C19—C20	1.403 (12)	C27"—C28"	1.378 (14)
C20—H20	0.9500	C27"—C34"	1.537 (14)
C21—C22	1.396 (12)	C27'—H27'	1.0000
C21—C26	1.393 (12)	C27'—C28'	1.376 (14)
C22—H22	0.9500	C27'—C34'	1.536 (14)
C22—C23	1.400 (12)	C28"—H28"	1.0000
C23—H23	0.9500	C28"—C29"	1.539 (14)
C23—C24	1.382 (12)	C28'—H28'	1.0000
C24—H24	0.9500	C28'—C29'	1.544 (14)
C24—C25	1.389 (12)	C29"—H29A	0.9900
C25—H25	0.9500	C29"—H29B	0.9900
C25—C26	1.388 (12)	C29"—C30"	1.548 (14)
C26—H26	0.9500	C29'—H29C	0.9900
C27—H27	1.0000	C29'—H29D	0.9900
C27—C28	1.378 (13)	C29'—C30'	1.543 (14)
C27—C34	1.535 (12)	C30"—H30A	0.9900
C28—H28	1.0000	C30"—H30B	0.9900
C28—C29	1.527 (12)	C30"—C31"	1.532 (14)
C29—H29E	0.9900	C30'—H30C	0.9900
C29—H29F	0.9900	C30'—H30D	0.9900
C29—C30	1.537 (13)	C30'—C31'	1.540 (14)
C30—H30E	0.9900	C31"—H31"	1.0000
C30—H30F	0.9900	C31"—C32"	1.378 (14)
C30—C31	1.537 (13)	C31'—H31'	1.0000
C31—H31	1.0000	C31'—C32'	1.380 (14)
C31—C32	1.372 (13)	C32"—H32"	1.0000
C32—H32	1.0000	C32"—C33"	1.537 (14)
C32—C33	1.538 (13)	C32'—H32'	1.0000
C33—H33E	0.9900	C32'—C33'	1.540 (14)
C33—H33F	0.9900	C33"—H33A	0.9900
C33—C34	1.537 (13)	C33"—H33B	0.9900
C34—H34E	0.9900	C33"—C34"	1.542 (14)
C34—H34F	0.9900	C33'—H33C	0.9900
Ir1'—P1'	2.329 (4)	C33'—H33D	0.9900
Ir1'—C1'	2.029 (12)	C33'—C34'	1.540 (14)
Ir1'—C27"	2.23 (5)	C34"—H34A	0.9900
Ir1'—C27'	2.21 (5)	C34"—H34B	0.9900
Ir1'—C28"	2.17 (5)	C34'—H34C	0.9900
Ir1'—C28'	2.27 (5)	C34'—H34D	0.9900
Ir1'—C31"	2.12 (3)	Cl1—C35	1.75 (4)
Ir1'—C31'	2.15 (3)	Cl2—C35	1.64 (4)
Ir1'—C32"	2.24 (3)	C35—H35A	0.9900
Ir1'—C32'	2.20 (3)	C35—H35B	0.9900
P1'—C9'	1.824 (14)	F1—B1	1.37 (3)
P1'—C15'	1.825 (9)	F2—B1	1.35 (3)
P1'—C21'	1.807 (14)	F3—B1	1.39 (3)
N1'—N2'	1.379 (9)	F4—B1	1.38 (3)
N1'—C1'	1.374 (12)	F5—B2	1.40 (3)
N1'—C3'	1.442 (12)	F6—B2	1.40 (3)
N2'—C2'	1.276 (12)	F7—B2	1.38 (3)
N3'—C1'	1.370 (12)	F8—B2	1.38 (3)
			
C1—Ir1—P1	93.5 (5)	C1'—N3'—C5'	124.2 (12)
C1—Ir1—C27	166.9 (5)	C2'—N3'—C5'	127.2 (12)
C1—Ir1—C28	154.0 (5)	N1'—C1'—Ir1'	125.6 (9)
C1—Ir1—C31	87.6 (6)	N3'—C1'—Ir1'	130.6 (9)
C1—Ir1—C32	87.3 (6)	N3'—C1'—N1'	103.7 (10)
C27—Ir1—P1	92.1 (4)	N2'—C2'—N3'	110.4 (13)
C27—Ir1—C31	88.3 (6)	N2'—C2'—H2'	124.8
C28—Ir1—P1	95.5 (4)	N3'—C2'—H2'	124.8
C28—Ir1—C27	36.4 (4)	N1'—C3'—H3'A	109.1
C28—Ir1—C31	80.6 (5)	N1'—C3'—H3'B	109.1
C31—Ir1—P1	172.9 (4)	N1'—C3'—C4'	112.4 (12)
C32—Ir1—P1	150.9 (4)	H3'A—C3'—H3'B	107.9
C32—Ir1—C27	82.1 (6)	C4'—C3'—H3'A	109.1
C32—Ir1—C28	96.3 (6)	C4'—C3'—H3'B	109.1
C32—Ir1—C31	36.1 (4)	C3'—C4'—H4'A	109.5
C9—P1—Ir1	109.7 (4)	C3'—C4'—H4'B	109.5
C9—P1—C15	102.4 (6)	C3'—C4'—H4'C	109.5
C9—P1—C21	103.7 (6)	H4'A—C4'—H4'B	109.5
C15—P1—Ir1	118.4 (4)	H4'A—C4'—H4'C	109.5
C21—P1—Ir1	115.0 (5)	H4'B—C4'—H4'C	109.5
C21—P1—C15	105.8 (6)	N3'—C5'—H5'A	108.6
N2—N1—C3	121.6 (11)	N3'—C5'—H5'B	108.6
C1—N1—N2	112.0 (10)	N3'—C5'—C6'	114.5 (13)
C1—N1—C3	126.4 (11)	H5'A—C5'—H5'B	107.6
C2—N2—N1	104.4 (11)	C6'—C5'—H5'A	108.6
C1—N3—C2	107.4 (11)	C6'—C5'—H5'B	108.6
C1—N3—C5	124.1 (11)	C5'—C6'—H6'	108.4
C2—N3—C5	128.2 (12)	C7'—C6'—C5'	110.2 (15)
N1—C1—Ir1	124.0 (9)	C7'—C6'—H6'	108.4
N1—C1—N3	103.9 (10)	C7'—C6'—C8'	113.1 (18)
N3—C1—Ir1	132.0 (9)	C8'—C6'—C5'	108.2 (14)
N2—C2—N3	112.2 (12)	C8'—C6'—H6'	108.4
N2—C2—H2	123.9	C6'—C7'—H7'A	109.5
N3—C2—H2	123.9	C6'—C7'—H7'B	109.5
N1—C3—H3A	109.6	C6'—C7'—H7'C	109.5
N1—C3—H3B	109.6	H7'A—C7'—H7'B	109.5
N1—C3—C4	110.4 (12)	H7'A—C7'—H7'C	109.5
H3A—C3—H3B	108.1	H7'B—C7'—H7'C	109.5
C4—C3—H3A	109.6	C6'—C8'—H8'A	109.5
C4—C3—H3B	109.6	C6'—C8'—H8'B	109.5
C3—C4—H4A	109.5	C6'—C8'—H8'C	109.5
C3—C4—H4B	109.5	H8'A—C8'—H8'B	109.5
C3—C4—H4C	109.5	H8'A—C8'—H8'C	109.5
H4A—C4—H4B	109.5	H8'B—C8'—H8'C	109.5
H4A—C4—H4C	109.5	C10'—C9'—P1'	117.9 (9)
H4B—C4—H4C	109.5	C10'—C9'—C14'	117.5 (13)
N3—C5—H5A	108.9	C14'—C9'—P1'	124.3 (10)
N3—C5—H5B	108.9	C9'—C10'—H10'	118.9
N3—C5—C6	113.5 (13)	C11'—C10'—C9'	122.2 (13)
H5A—C5—H5B	107.7	C11'—C10'—H10'	118.9
C6—C5—H5A	108.9	C10'—C11'—H11'	119.9
C6—C5—H5B	108.9	C12'—C11'—C10'	120.2 (14)
C5—C6—H6	108.9	C12'—C11'—H11'	119.9
C5—C6—C7	111.4 (16)	C11'—C12'—H12'	120.8
C5—C6—C8	108.7 (13)	C11'—C12'—C13'	118.5 (13)
C7—C6—H6	108.9	C13'—C12'—H12'	120.8
C8—C6—H6	108.9	C12'—C13'—H13'	119.4
C8—C6—C7	110 (2)	C14'—C13'—C12'	121.2 (14)
C6—C7—H7A	109.5	C14'—C13'—H13'	119.4
C6—C7—H7B	109.5	C9'—C14'—H14'	119.8
C6—C7—H7C	109.5	C13'—C14'—C9'	120.3 (14)
H7A—C7—H7B	109.5	C13'—C14'—H14'	119.8
H7A—C7—H7C	109.5	C16'—C15'—P1'	120.5 (6)
H7B—C7—H7C	109.5	C16'—C15'—C20'	120.0
C6—C8—H8A	109.5	C20'—C15'—P1'	119.5 (6)
C6—C8—H8B	109.5	C15'—C16'—H16'	120.0
C6—C8—H8C	109.5	C17'—C16'—C15'	120.0
H8A—C8—H8B	109.5	C17'—C16'—H16'	120.0
H8A—C8—H8C	109.5	C16'—C17'—H17'	120.0
H8B—C8—H8C	109.5	C18'—C17'—C16'	120.0
C10—C9—P1	120.2 (10)	C18'—C17'—H17'	120.0
C14—C9—P1	120.0 (10)	C17'—C18'—H18'	120.0
C14—C9—C10	119.8 (13)	C17'—C18'—C19'	120.0
C9—C10—H10	120.3	C19'—C18'—H18'	120.0
C11—C10—C9	119.5 (14)	C18'—C19'—H19'	120.0
C11—C10—H10	120.3	C18'—C19'—C20'	120.0
C10—C11—H11	119.3	C20'—C19'—H19'	120.0
C10—C11—C12	121.4 (15)	C15'—C20'—H20'	120.0
C12—C11—H11	119.3	C19'—C20'—C15'	120.0
C11—C12—H12	120.8	C19'—C20'—H20'	120.0
C11—C12—C13	118.5 (15)	C22'—C21'—P1'	123.6 (10)
C13—C12—H12	120.8	C26'—C21'—P1'	118.5 (10)
C12—C13—H13	119.4	C26'—C21'—C22'	117.5 (13)
C12—C13—C14	121.2 (15)	C21'—C22'—H22'	119.6
C14—C13—H13	119.4	C23'—C22'—C21'	120.8 (14)
C9—C14—C13	119.5 (14)	C23'—C22'—H22'	119.6
C9—C14—H14	120.2	C22'—C23'—H23'	119.9
C13—C14—H14	120.2	C24'—C23'—C22'	120.3 (14)
C16—C15—P1	117.6 (9)	C24'—C23'—H23'	119.9
C20—C15—P1	124.1 (9)	C23'—C24'—H24'	120.0
C20—C15—C16	118.2 (13)	C23'—C24'—C25'	120.1 (14)
C15—C16—H16	118.6	C25'—C24'—H24'	120.0
C17—C16—C15	122.8 (13)	C24'—C25'—H25'	120.7
C17—C16—H16	118.6	C26'—C25'—C24'	118.6 (14)
C16—C17—H17	121.0	C26'—C25'—H25'	120.7
C16—C17—C18	118.0 (14)	C21'—C26'—H26'	118.7
C18—C17—H17	121.0	C25'—C26'—C21'	122.6 (14)
C17—C18—H18	119.8	C25'—C26'—H26'	118.7
C19—C18—C17	120.5 (14)	Ir1'—C27"—H27"	115.2
C19—C18—H18	119.8	C28"—C27"—Ir1'	70 (3)
C18—C19—H19	120.0	C28"—C27"—H27"	115.2
C18—C19—C20	120.1 (13)	C28"—C27"—C34"	116 (3)
C20—C19—H19	120.0	C34"—C27"—Ir1'	118 (3)
C15—C20—C19	120.3 (13)	C34"—C27"—H27"	115.2
C15—C20—H20	119.8	Ir1'—C27'—H27'	113.8
C19—C20—H20	119.8	C28'—C27'—Ir1'	74 (3)
C22—C21—P1	118.1 (9)	C28'—C27'—H27'	113.8
C26—C21—P1	122.2 (9)	C28'—C27'—C34'	128 (4)
C26—C21—C22	119.6 (13)	C34'—C27'—Ir1'	103 (2)
C21—C22—H22	120.5	C34'—C27'—H27'	113.8
C21—C22—C23	119.1 (13)	Ir1'—C28"—H28"	114.7
C23—C22—H22	120.5	C27"—C28"—Ir1'	74 (3)
C22—C23—H23	119.6	C27"—C28"—H28"	114.7
C24—C23—C22	120.9 (13)	C27"—C28"—C29"	125 (4)
C24—C23—H23	119.6	C29"—C28"—Ir1'	106 (3)
C23—C24—H24	120.0	C29"—C28"—H28"	114.7
C23—C24—C25	120.0 (13)	Ir1'—C28'—H28'	114.2
C25—C24—H24	120.0	C27'—C28'—Ir1'	70 (2)
C24—C25—H25	120.2	C27'—C28'—H28'	114.2
C26—C25—C24	119.6 (13)	C27'—C28'—C29'	125 (4)
C26—C25—H25	120.2	C29'—C28'—Ir1'	111 (3)
C21—C26—H26	119.6	C29'—C28'—H28'	114.2
C25—C26—C21	120.9 (13)	C28"—C29"—H29A	108.4
C25—C26—H26	119.6	C28"—C29"—H29B	108.4
Ir1—C27—H27	114.1	C28"—C29"—C30"	116 (3)
C28—C27—Ir1	70.9 (10)	H29A—C29"—H29B	107.4
C28—C27—H27	114.1	C30"—C29"—H29A	108.4
C28—C27—C34	124.5 (15)	C30"—C29"—H29B	108.4
C34—C27—Ir1	111.3 (11)	C28'—C29'—H29C	108.7
C34—C27—H27	114.1	C28'—C29'—H29D	108.7
Ir1—C28—H28	113.6	H29C—C29'—H29D	107.6
C27—C28—Ir1	72.7 (10)	C30'—C29'—C28'	114 (3)
C27—C28—H28	113.6	C30'—C29'—H29C	108.7
C27—C28—C29	126.5 (14)	C30'—C29'—H29D	108.7
C29—C28—Ir1	109.0 (10)	C29"—C30"—H30A	109.7
C29—C28—H28	113.6	C29"—C30"—H30B	109.7
C28—C29—H29E	108.9	H30A—C30"—H30B	108.2
C28—C29—H29F	108.9	C31"—C30"—C29"	110 (3)
C28—C29—C30	113.3 (13)	C31"—C30"—H30A	109.7
H29E—C29—H29F	107.7	C31"—C30"—H30B	109.7
C30—C29—H29E	108.9	C29'—C30'—H30C	109.8
C30—C29—H29F	108.9	C29'—C30'—H30D	109.8
C29—C30—H30E	109.2	H30C—C30'—H30D	108.2
C29—C30—H30F	109.2	C31'—C30'—C29'	110 (3)
C29—C30—C31	112.2 (13)	C31'—C30'—H30C	109.8
H30E—C30—H30F	107.9	C31'—C30'—H30D	109.8
C31—C30—H30E	109.2	Ir1'—C31"—H31"	113.0
C31—C30—H30F	109.2	C30"—C31"—Ir1'	115 (2)
Ir1—C31—H31	114.4	C30"—C31"—H31"	113.0
C30—C31—Ir1	111.9 (11)	C32"—C31"—Ir1'	76.5 (19)
C30—C31—H31	114.4	C32"—C31"—C30"	122 (3)
C32—C31—Ir1	70.3 (10)	C32"—C31"—H31"	113.0
C32—C31—C30	123.6 (16)	Ir1'—C31'—H31'	110.7
C32—C31—H31	114.4	C30'—C31'—Ir1'	113 (2)
Ir1—C32—H32	114.3	C30'—C31'—H31'	110.7
C31—C32—Ir1	73.5 (10)	C32'—C31'—Ir1'	73.7 (19)
C31—C32—H32	114.3	C32'—C31'—C30'	131 (3)
C31—C32—C33	125.2 (16)	C32'—C31'—H31'	110.7
C33—C32—Ir1	107.1 (11)	Ir1'—C32"—H32"	114.4
C33—C32—H32	114.3	C31"—C32"—Ir1'	66.8 (18)
C32—C33—H33E	108.6	C31"—C32"—H32"	114.4
C32—C33—H33F	108.6	C31"—C32"—C33"	125 (3)
H33E—C33—H33F	107.5	C33"—C32"—Ir1'	112 (2)
C34—C33—C32	114.8 (14)	C33"—C32"—H32"	114.4
C34—C33—H33E	108.6	Ir1'—C32'—H32'	115.6
C34—C33—H33F	108.6	C31'—C32'—Ir1'	69.4 (19)
C27—C34—C33	113.0 (14)	C31'—C32'—H32'	115.6
C27—C34—H34E	109.0	C31'—C32'—C33'	121 (3)
C27—C34—H34F	109.0	C33'—C32'—Ir1'	110 (2)
C33—C34—H34E	109.0	C33'—C32'—H32'	115.6
C33—C34—H34F	109.0	C32"—C33"—H33A	109.0
H34E—C34—H34F	107.8	C32"—C33"—H33B	109.0
C1'—Ir1'—P1'	93.4 (4)	C32"—C33"—C34"	113 (3)
C1'—Ir1'—C27"	155.3 (9)	H33A—C33"—H33B	107.8
C1'—Ir1'—C27'	163.2 (8)	C34"—C33"—H33A	109.0
C1'—Ir1'—C28"	165.6 (9)	C34"—C33"—H33B	109.0
C1'—Ir1'—C28'	158.2 (9)	C32'—C33'—H33C	109.2
C1'—Ir1'—C31"	89.3 (9)	C32'—C33'—H33D	109.2
C1'—Ir1'—C31'	85.3 (9)	H33C—C33'—H33D	107.9
C1'—Ir1'—C32"	86.3 (9)	C34'—C33'—C32'	112 (2)
C1'—Ir1'—C32'	87.4 (9)	C34'—C33'—H33C	109.2
C27"—Ir1'—P1'	97.3 (9)	C34'—C33'—H33D	109.2
C27"—Ir1'—C32"	76.6 (11)	C27"—C34"—C33"	107 (2)
C27'—Ir1'—P1'	94.7 (10)	C27"—C34"—H34A	110.3
C27'—Ir1'—C28'	35.8 (7)	C27"—C34"—H34B	110.3
C28"—Ir1'—P1'	90.9 (10)	C33"—C34"—H34A	110.3
C28"—Ir1'—C27"	36.5 (7)	C33"—C34"—H34B	110.3
C28"—Ir1'—C32"	93.9 (13)	H34A—C34"—H34B	108.6
C28'—Ir1'—P1'	92.4 (10)	C27'—C34'—C33'	115 (3)
C31"—Ir1'—P1'	161.5 (7)	C27'—C34'—H34C	108.6
C31"—Ir1'—C27"	87.6 (12)	C27'—C34'—H34D	108.6
C31"—Ir1'—C28"	82.4 (12)	C33'—C34'—H34C	108.6
C31"—Ir1'—C32"	36.7 (5)	C33'—C34'—H34D	108.6
C31'—Ir1'—P1'	149.7 (7)	H34C—C34'—H34D	107.6
C31'—Ir1'—C27'	95.0 (13)	Cl1—C35—H35A	109.0
C31'—Ir1'—C28'	79.2 (11)	Cl1—C35—H35B	109.0
C31'—Ir1'—C32'	36.9 (5)	Cl2—C35—Cl1	113 (2)
C32"—Ir1'—P1'	161.8 (7)	Cl2—C35—H35A	109.0
C32'—Ir1'—P1'	173.4 (7)	Cl2—C35—H35B	109.0
C32'—Ir1'—C27'	82.9 (11)	H35A—C35—H35B	107.8
C32'—Ir1'—C28'	89.3 (12)	F1—B1—F3	110.0 (17)
C9'—P1'—Ir1'	117.5 (5)	F1—B1—F4	108 (2)
C9'—P1'—C15'	101.7 (6)	F2—B1—F1	110.9 (18)
C15'—P1'—Ir1'	112.7 (4)	F2—B1—F3	110 (2)
C21'—P1'—Ir1'	114.2 (5)	F2—B1—F4	109.1 (19)
C21'—P1'—C9'	105.6 (6)	F4—B1—F3	109.1 (18)
C21'—P1'—C15'	103.5 (6)	F6—B2—F5	110.2 (18)
N2'—N1'—C3'	121.2 (11)	F7—B2—F5	109 (2)
C1'—N1'—N2'	110.8 (10)	F7—B2—F6	109.4 (19)
C1'—N1'—C3'	128.0 (11)	F7—B2—F8	108.7 (19)
C2'—N2'—N1'	106.7 (12)	F8—B2—F5	109.6 (19)
C1'—N3'—C2'	108.4 (11)	F8—B2—F6	109 (2)
			
Ir1—P1—C9—C10	18.4 (13)	Ir1'—C27'—C28'—C29'	−102 (4)
Ir1—P1—C9—C14	−162.1 (11)	Ir1'—C27'—C34'—C33'	45 (3)
Ir1—P1—C15—C16	64.3 (12)	Ir1'—C28"—C29"—C30"	−38 (4)
Ir1—P1—C15—C20	−114.2 (12)	Ir1'—C28'—C29'—C30'	14 (4)
Ir1—P1—C21—C22	69.9 (12)	Ir1'—C31"—C32"—C33"	−102 (3)
Ir1—P1—C21—C26	−106.6 (11)	Ir1'—C31'—C32'—C33'	−102 (3)
Ir1—C27—C28—C29	−101.2 (17)	Ir1'—C32"—C33"—C34"	−36 (3)
Ir1—C27—C34—C33	−10.0 (18)	Ir1'—C32'—C33'—C34'	13 (3)
Ir1—C28—C29—C30	−39.7 (16)	P1'—C9'—C10'—C11'	175.7 (13)
Ir1—C31—C32—C33	−99.5 (17)	P1'—C9'—C14'—C13'	−174.7 (13)
Ir1—C32—C33—C34	−38.3 (18)	P1'—C15'—C16'—C17'	−177.3 (9)
P1—C9—C10—C11	178.4 (13)	P1'—C15'—C20'—C19'	177.3 (9)
P1—C9—C14—C13	−177.1 (13)	P1'—C21'—C22'—C23'	−174.8 (12)
P1—C15—C16—C17	−177.0 (13)	P1'—C21'—C26'—C25'	177.1 (12)
P1—C15—C20—C19	176.0 (12)	N1'—N2'—C2'—N3'	1.5 (18)
P1—C21—C22—C23	−177.0 (11)	N2'—N1'—C1'—Ir1'	−176.2 (11)
P1—C21—C26—C25	176.6 (11)	N2'—N1'—C1'—N3'	0.3 (17)
N1—N2—C2—N3	0 (2)	N2'—N1'—C3'—C4'	−71.5 (19)
N2—N1—C1—Ir1	179.2 (11)	N3'—C5'—C6'—C7'	−65 (2)
N2—N1—C1—N3	2.1 (17)	N3'—C5'—C6'—C8'	170.5 (17)
N2—N1—C3—C4	69.3 (18)	C1'—N1'—N2'—C2'	−1.2 (18)
N3—C5—C6—C7	63 (2)	C1'—N1'—C3'—C4'	109.0 (18)
N3—C5—C6—C8	−175.6 (19)	C1'—N3'—C2'—N2'	−1.4 (19)
C1—N1—N2—C2	−1.2 (19)	C1'—N3'—C5'—C6'	137.4 (16)
C1—N1—C3—C4	−108.4 (17)	C2'—N3'—C1'—Ir1'	176.9 (12)
C1—N3—C2—N2	2 (2)	C2'—N3'—C1'—N1'	0.6 (17)
C1—N3—C5—C6	−140.6 (17)	C2'—N3'—C5'—C6'	−49 (2)
C2—N3—C1—Ir1	−178.9 (13)	C3'—N1'—N2'—C2'	179.2 (14)
C2—N3—C1—N1	−2.1 (17)	C3'—N1'—C1'—Ir1'	3 (2)
C2—N3—C5—C6	47 (3)	C3'—N1'—C1'—N3'	179.9 (14)
C3—N1—N2—C2	−179.2 (15)	C5'—N3'—C1'—Ir1'	−8 (2)
C3—N1—C1—Ir1	−3 (2)	C5'—N3'—C1'—N1'	175.7 (14)
C3—N1—C1—N3	180.0 (14)	C5'—N3'—C2'—N2'	−176.3 (15)
C5—N3—C1—Ir1	7 (2)	C9'—P1'—C15'—C16'	−146.5 (7)
C5—N3—C1—N1	−176.0 (14)	C9'—P1'—C15'—C20'	36.2 (8)
C5—N3—C2—N2	175.2 (16)	C9'—P1'—C21'—C22'	−119.8 (13)
C9—P1—C15—C16	−56.5 (12)	C9'—P1'—C21'—C26'	67.3 (13)
C9—P1—C15—C20	125.0 (13)	C9'—C10'—C11'—C12'	−3 (3)
C9—P1—C21—C22	−170.2 (11)	C10'—C9'—C14'—C13'	−1 (2)
C9—P1—C21—C26	13.3 (13)	C10'—C11'—C12'—C13'	4 (2)
C9—C10—C11—C12	0 (3)	C11'—C12'—C13'—C14'	−4 (2)
C10—C9—C14—C13	2 (2)	C12'—C13'—C14'—C9'	2 (2)
C10—C11—C12—C13	−1 (3)	C14'—C9'—C10'—C11'	1 (2)
C11—C12—C13—C14	2 (3)	C15'—P1'—C9'—C10'	59.5 (13)
C12—C13—C14—C9	−3 (3)	C15'—P1'—C9'—C14'	−126.5 (14)
C14—C9—C10—C11	−1 (2)	C15'—P1'—C21'—C22'	−13.4 (14)
C15—P1—C9—C10	145.1 (12)	C15'—P1'—C21'—C26'	173.7 (11)
C15—P1—C9—C14	−35.5 (14)	C15'—C16'—C17'—C18'	0.0
C15—P1—C21—C22	−62.8 (13)	C16'—C15'—C20'—C19'	0.0
C15—P1—C21—C26	120.7 (12)	C16'—C17'—C18'—C19'	0.0
C15—C16—C17—C18	1 (2)	C17'—C18'—C19'—C20'	0.0
C16—C15—C20—C19	−2 (2)	C18'—C19'—C20'—C15'	0.0
C16—C17—C18—C19	−2 (3)	C20'—C15'—C16'—C17'	0.0
C17—C18—C19—C20	2 (3)	C21'—P1'—C9'—C10'	167.3 (12)
C18—C19—C20—C15	1 (2)	C21'—P1'—C9'—C14'	−18.7 (15)
C20—C15—C16—C17	2 (2)	C21'—P1'—C15'—C16'	104.1 (8)
C21—P1—C9—C10	−105.0 (12)	C21'—P1'—C15'—C20'	−73.2 (8)
C21—P1—C9—C14	74.5 (14)	C21'—C22'—C23'—C24'	0 (2)
C21—P1—C15—C16	−164.8 (11)	C22'—C21'—C26'—C25'	4 (2)
C21—P1—C15—C20	16.6 (14)	C22'—C23'—C24'—C25'	1 (2)
C21—C22—C23—C24	1 (2)	C23'—C24'—C25'—C26'	1 (2)
C22—C21—C26—C25	0 (2)	C24'—C25'—C26'—C21'	−3 (2)
C22—C23—C24—C25	0 (2)	C26'—C21'—C22'—C23'	−2 (2)
C23—C24—C25—C26	0 (2)	C27"—C28"—C29"—C30"	43 (6)
C24—C25—C26—C21	0 (2)	C27'—C28'—C29'—C30'	94 (5)
C26—C21—C22—C23	0 (2)	C28"—C27"—C34"—C33"	−106 (4)
C27—C28—C29—C30	42 (2)	C28"—C29"—C30"—C31"	29 (5)
C28—C27—C34—C33	−91 (2)	C28'—C27'—C34'—C33'	−35 (6)
C28—C29—C30—C31	37 (2)	C28'—C29'—C30'—C31'	−34 (4)
C29—C30—C31—Ir1	−15.1 (18)	C29"—C30"—C31"—Ir1'	−5 (4)
C29—C30—C31—C32	−95 (2)	C29"—C30"—C31"—C32"	−94 (4)
C30—C31—C32—Ir1	103.7 (16)	C29'—C30'—C31'—Ir1'	40 (3)
C30—C31—C32—C33	4 (3)	C29'—C30'—C31'—C32'	−49 (5)
C31—C32—C33—C34	43 (2)	C30"—C31"—C32"—Ir1'	111 (3)
C32—C33—C34—C27	33 (2)	C30"—C31"—C32"—C33"	9 (5)
C34—C27—C28—Ir1	103.1 (16)	C30'—C31'—C32'—Ir1'	107 (4)
C34—C27—C28—C29	2 (3)	C30'—C31'—C32'—C33'	5 (6)
Ir1'—P1'—C9'—C10'	−64.1 (13)	C31"—C32"—C33"—C34"	40 (4)
Ir1'—P1'—C9'—C14'	110.0 (13)	C31'—C32'—C33'—C34'	91 (4)
Ir1'—P1'—C15'—C16'	−19.7 (8)	C32"—C33"—C34"—C27"	40 (4)
Ir1'—P1'—C15'—C20'	162.9 (5)	C32'—C33'—C34'—C27'	−41 (4)
Ir1'—P1'—C21'—C22'	109.6 (12)	C34"—C27"—C28"—Ir1'	112 (3)
Ir1'—P1'—C21'—C26'	−63.4 (12)	C34"—C27"—C28"—C29"	13 (6)
Ir1'—C27"—C28"—C29"	−98 (4)	C34'—C27'—C28'—Ir1'	95 (4)
Ir1'—C27"—C34"—C33"	−26 (4)	C34'—C27'—C28'—C29'	−8 (7)

### [(1,2,5,6-η)-Cycloocta-1,5-diene](1-ethyl-4-isobutyl-1,2,4-triazol-5-ylidene)(triphenylphosphane)iridium(I) tetrafluoridoborate dichloromethane hemisolvate Hydrogen-bond geometry (Å, º)

**Table:**

*D*—H···*A*	*D*—H	H···*A*	*D*···*A*	*D*—H···*A*
C2—H2···F8^i^	0.95	2.32	3.18 (2)	149
C19—H19···F1^ii^	0.95	2.50	3.380 (19)	154
C2′—H2′···F5^iii^	0.95	2.26	3.171 (18)	161
C13′—H13′···F2^iii^	0.95	2.56	3.38 (2)	144
C18′—H18′···F8	0.95	2.52	3.319 (16)	142
C33"—H33*B*···F3^ii^	0.99	2.24	3.08 (3)	143

Symmetry codes: (i) *x*−1/2, −*y*+3/2, *z*−1/2; (ii) *x*−1, *y*, *z*; (iii) *x*−1/2, *y*−1/2, *z*.
